# Transformer-based framework for multi-class segmentation of skin cancer from histopathology images

**DOI:** 10.3389/fmed.2024.1380405

**Published:** 2024-04-29

**Authors:** Muhammad Imran, Mohsin Islam Tiwana, Mashood Mohammad Mohsan, Norah Saleh Alghamdi, Muhammad Usman Akram

**Affiliations:** ^1^Department of Mechatronics Engineering, National University of Sciences and Technology, Islamabad, Pakistan; ^2^Department of Computer and Software Engineering, National University of Sciences and Technology, Islamabad, Pakistan; ^3^Department of Computer Sciences, Princess Nourah Bint Abdulrahman University, Riyadh, Saudi Arabia

**Keywords:** segmentation, histology image analysis, semantic segmentation, image transformers, deep learning

## Abstract

**Introduction:**

Non-melanoma skin cancer comprising Basal cell carcinoma (BCC), Squamous cell carcinoma (SCC), and Intraepidermal carcinoma (IEC) has the highest incidence rate among skin cancers. Intelligent decision support systems may address the issue of the limited number of subject experts and help in mitigating the parity of health services between urban centers and remote areas.

**Method:**

In this research, we propose a transformer-based model for the segmentation of histopathology images not only into inflammation and cancers such as BCC, SCC, and IEC but also to identify skin tissues and boundaries that are important in decision-making. Accurate segmentation of these tissue types will eventually lead to accurate detection and classification of non-melanoma skin cancer. The segmentation according to tissue types and their visual representation before classification enhances the trust of pathologists and doctors being relatable to how most pathologists approach this problem. The visualization of the confidence of the model in its prediction through uncertainty maps is also what distinguishes this study from most deep learning methods.

**Results:**

The evaluation of proposed system is carried out using publicly available dataset. The application of our proposed segmentation system demonstrated good performance with an F1 score of 0.908, mean intersection over union (mIoU) of 0.653, and average accuracy of 83.1%, advocating that the system can be used as a decision support system successfully and has the potential of subsequently maturing into a fully automated system.

**Discussion:**

This study is an attempt to automate the segmentation of the most occurring non-melanoma skin cancer using a transformer-based deep learning technique applied to histopathology skin images. Highly accurate segmentation and visual representation of histopathology images according to tissue types by the proposed system implies that the system can be used for skin-related routine pathology tasks including cancer and other anomaly detection, their classification, and measurement of surgical margins in the case of cancer cases.

## 1 Introduction

### 1.1 Skin cancer: a global health challenge

Skin cancer, particularly non-melanoma skin cancer (NMSC), is a global health challenge with consistently rising incidence rates. The study evaluating the Global Burden of Disease (GBD) for 22 different types of diseases and injuries in 204 countries and territories of the world reports cancer to be the deadliest disease after cardiovascular diseases for the year 2019. The study reports 23.6 million new cancer cases and 10 million cancer deaths in the year 2019 (1). An earlier study reports 24.5 million cases of cancer and 9.6 million deaths worldwide in 2017, along with 7.7 million reported cases of non-melanoma skin cancer (NMSC). Basal cell carcinoma (BCC) and squamous cell carcinoma (SCC) accounted for 5.9 million and 1.8 million cases, respectively. The incidence of NMSC increased by 33% between 2007 and 2017 from 5.8 million to 7.7 million cases (2). Moreover, non-melanoma skin cancers account for more than 98% of all skin cancers in the United States where BCC and SCC are numbered 2.8 million and 1.5 million, respectively, in comparison to 82,054 cases of melanoma in 2019 (3). Intraepidermal carcinoma (IEC), also known as squamous carcinoma *in situ* or Bowen's disease, is another but less occurring type of non-melanoma skin cancer. It is, however, found that ~3–5% of IEC cases evolve into invasive squamous cell carcinoma (4).

The latest research in recent years has widely demonstrated that many diagnostic tasks in the medical field can be successfully assisted or performed independently by AI using deep learning, convolutional neural networks, and machine learning algorithms (5–7). AI and digital processing techniques have significantly improved the accuracy and speed of medical diagnosis. These advancements are not only limited to the analysis of images but also include the processing of signals such as ECGs. Advancements in healthcare are multifaceted. Post-processing of CT images for improved image quality and reduced radiation dose (8), denoising of ECG signals for enhanced signal clarity (9), clustering-based multi-modality image fusion to minimize noise (10), and advanced diagnosis and detection of lung diseases (11) are some of the transformative contributions of AI and digital processing techniques in medical care. These innovations collectively mark significant progress in improving healthcare outcomes. Progress of a similar scale has also been made in the field of digital pathology (12, 13). It is worth noting that while AI can aid in detecting and diagnosing cancers, it is not yet advanced enough to replace radiologists and pathologists in medical image analysis (14). With the probability of an impending shortage of pathologists in the future (15), automated techniques are becoming essentially required for relieving pathologists for more complex tasks. These automated AI techniques can also help in eliminating human-induced bias from diagnosis besides assisting them with their job.

As skin cancer, particularly non-melanoma skin cancer (NMSC), poses a global health challenge as per the above-cited facts, this research aims to develop an accurate segmentation technique for identifying various skin tissues, inflammations, and carcinomas from histopathology images. As BCC and SCC account for 98% of NMSC cases, the proposed method holds the potential to automate numerous skin cancer segmentation tasks, leading to expedited diagnosis and early detection.

### 1.2 Diagnosis of skin cancer

Many different histomorphological features on various tissue sections are evaluated through multiple slides by a pathologist while investigating the diagnosis of skin cancer. Evaluation of multiple slides is necessitated because the way samples are prepared for histopathology analysis directly affects the performance of the pathologist. The quality and accuracy of diagnosis and subsequent clinical interventions by physicians are also contingent upon the processing of samples. This consequently warrants careful processing of samples; therefore, orientation of excision samples, irrespective of the way they are extracted, is preserved during sample processing and examinations with respect to placement on the patient. Blue and black inks are used to preserve orientation and indicate different margins. Specimens are sliced in cross sections of 3 mm thickness to have a detailed view and extent of surgical margins. These cross sections of the specimen are further treated with different solvents, saturated in paraffin, and further sliced into 3μ m transparent sections. These transparent micro slices are generally stained with hematoxylin and eosin (H & E) staining to make structures such as nuclei, cytoplasm, and stroma visibly differentiable (16–18). Finally, these processed and stained slides are analyzed and evaluated for finding lesions, determining their types in case they exist, the degree of certainty in diagnosis, and finding out the penetration in tissues. The study of slides also ascertains whether the excision has been complete. It also attempts to calculate surgical margins. The final diagnosis is reached based on all complementing and contradicting pieces of evidence found in the evaluation of all available slides. These diagnoses are also verified with available clinical history.

Consequently, we know that diagnosis of skin cancer is a compound task involving many complex sub-tasks. The Royal College of Pathologists (UK) has established a reporting protocol (19, 20), and pathologists are expected to prepare reports according to this protocol when a case of BCC, SCC, or any other lesion is detected. The job of pathologists is however more than described steps, and they are expected to identify cancer type, find evidence of lymphovascular or perineural involvement, and other aspects critical for subsequent treatment options. Cellular morphology, macroscopic characteristics, and growth patterns including depth of invasion are studied critically for identifying cancer type. Detection of lymphovascular and perineural involvement is important for considering clinical intervention for treatment; the aspect is more critical in case of metastasis. The investigation by pathologists and their reports put forth treatment options for oncologists and clinicians.

Citing the requirements of a useful pathology report discussed above, it is evident that a report must contain sufficient matter to help clinicians with their prognosis of condition and take the best-suited treatment option. As most of the previous work proposed for the digitization of skin cancer detection approach problem as a binary classification problem therefore has limited utility for clinicians in deploying these techniques in real cases. In addition, detection and diagnosis of skin cancer cases are critical tasks involving human life, therefore the way an algorithm reaches a particular decision and the certainty of correctness are also matters of concern both for clinicians and patients. Most binary classification approaches lack this transparency, whereas multi-class-semantic segmentation-based approaches later leading to detection and diagnosis are not only interpretable but also generate most parts of the report useful for clinicians.

### 1.3 AI and skin cancer diagnosis

In the recent past, many researchers have contributed to the field signifying how well AI can perform skin cancer detection and diagnosis tasks. A study published in 2017 demonstrated that a CNN model trained over 129,450 clinical images representing over 2,032 skin anomalies matched the performance of 21 certified dermatologists with an overall accuracy of 91% (21). A study employed Google's Inception V4 CNN architecture for the detection of skin lesions from dermoscopic images in comparison to 58 trained dermatologists. It manifested that the receiver operating characteristic area under the curve (ROC-AUC) achieved by the network was better than the mean ROC of dermatologists (22). Another study showed that a pre-trained ResNet50 convolutional neural network fine-tuned using 595 histopathology images can significantly outperform 11 pathologists of different expertise levels in the reading test set of 100 histopathology melanoma images (23). A similar study proposed an optimized deep-CNN architecture and modified mini-batch logic and loss function. The model was trained using 17,302 images to assess its performance with 157 dermatologists from 12 university hospitals in Germany. The study demonstrated that the proposed model outperformed all 157 dermatologists (24).

The cited studies compare AI with pathologists or dermatologists; however, a different study suggests combining AI and human experts. It finds out that AI and dermatologists while complementing each other can achieve superior performance as compared with their independent performances (25).

While so much research is being contributed to the field of AI-based skin cancer diagnosis, it is important to study the perception of patients regarding the use of AI in the diagnosis of their disease. A recent study, therefore, attempts to observe how patients perceive AI and its use for skin cancer screening. A sample of 48 patients with equal representation of melanoma, non-melanoma, and no skin cancer disease with a combination of men and women were part of the study. This qualitative study finds out that most patients consider higher diagnostic speed and access to healthcare as the main benefits of AI, and they would recommend AI to family and friends. They were, however, ambivalent about the accuracy of the diagnosis (26). Another similar study reaches out to 298 persons in a web-based survey and concludes that ~94% respondents agree to the use of AI in the medical field and 88% would even agree to anonymously provide their data for research, thus showing generally a receptive attitude toward AI in the field of medicine (27).

Pieces of evidence in preceding paras lead to a logical direction that AI approaches which are comprehendible and understandable for patients, pathologists, and clinicians have more likelihood of being implemented (28, 29). This is why many explainable and interpretable approaches for skin lesion detection have been introduced and are being aggressively pursued (30–35). The automatic segmentation of tissues, anomalies, and lesions in histopathology images can be a foundation stone for building an interpretable AI framework. Transformer-based approaches have lately shown great results in many areas including semantic segmentation (36) and seem promising for the future. This study, therefore, employs a novel transformer-based framework for multi-class segmentation of non-melanoma skin cancer using histopathology images. We evaluated the proposed framework for segmentation accuracy using three different performance measures and found it excellent considering the complexity of identifying 12 classes using limited data. In addition, we visually present the confidence of the segmentation model in its prediction through uncertainty maps to highlight areas of uncertainty and complexity in test samples. The contributions of our study are listed as follows:

- We propose a novel transformer-based framework for multi-class segmentation of skin tissues and non-melanoma skin cancer using one of the latest publicly available histopathology datasets.
- Through a series of experiments, we demonstrate the correlation of segmentation accuracy with the resolution of histopathology images. Moreover, we evaluate the performance of the proposed framework using three different performance measures and demonstrate its superior performance in segmenting 12 classes with limited data.
- We visually present the confidence of the model in its prediction through uncertainty maps, and these maps are of great help in highlighting areas of uncertainty and complexity in the specimen.

## 2 Method

### 2.1 Model architecture

**Figure 1:**
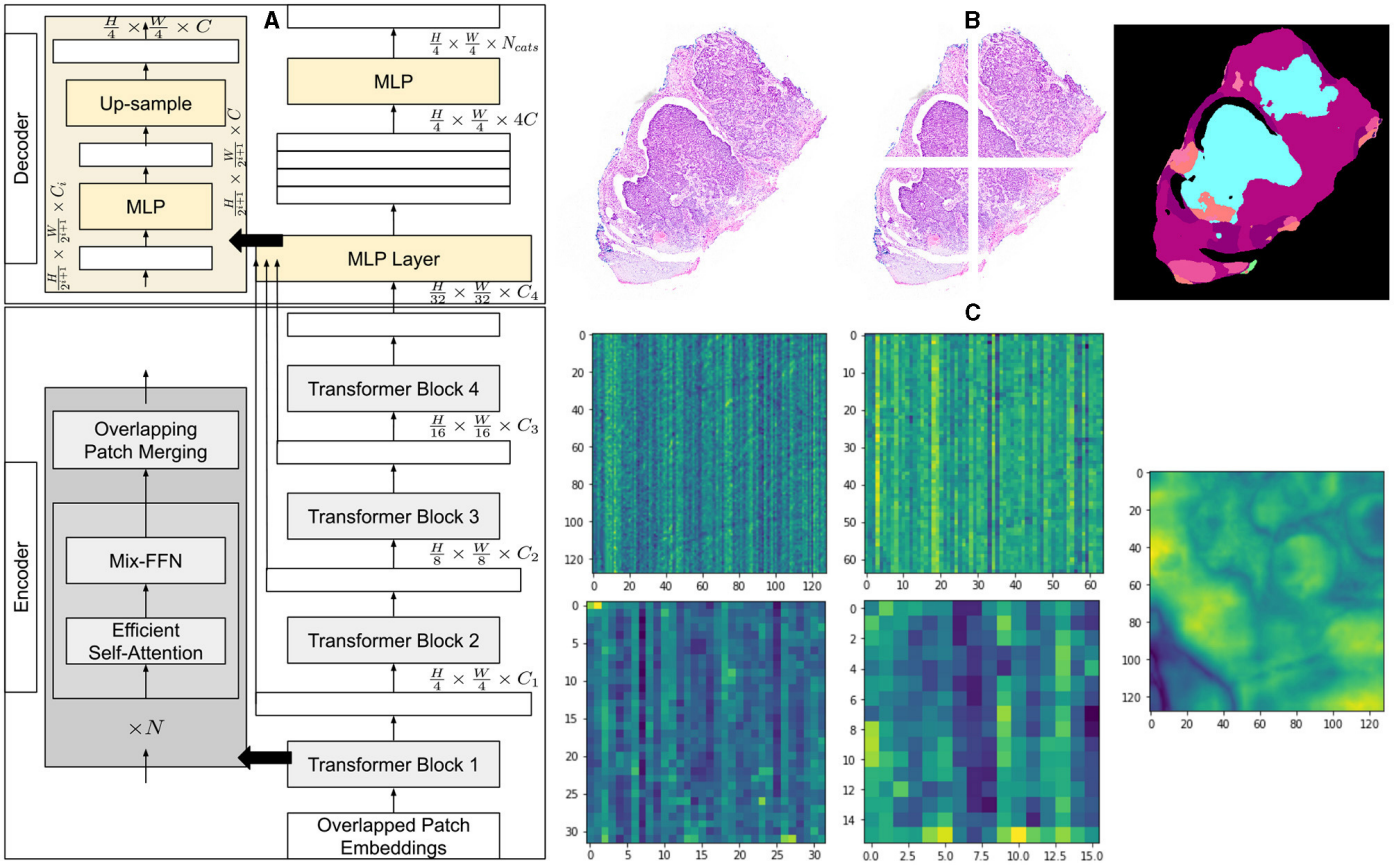
Architecture of segmentation model and overview of methodology. **(A)** A detailed explanation of the architecture of the segmentation model, illustrating the components of the encoder and decoder. **(B)** An example of a histopathology image with its corresponding patches and the segmented output of the model. **(C)** Visualization of the average feature maps extracted by each transformer block, along with the concatenated fusion of these features.

We employ a powerful, efficient, and robust segmentation framework (36) which is better suited to achieve a higher level of automation and is computationally inexpensive at the same time. Like most transformer-based frameworks, the architecture has two distinct modules, as shown in Figure 1. The first one is the encoder which is designed in a hierarchical way to extract coarse and fine features having high resolution and low resolution, respectively. The second module is a multi-layer perceptron (MLP) decoder that uses features extracted by the hierarchical encoder to output semantic segmentation of the input image.

The method first creates patches of 4 × 4 from the input image of size *H*×*W*×3. The smaller patch size of 4 × 4 has been found better suited for segmentation tasks with dense features. These patches of 4 × 4 are fed into a hierarchical encoder to extract four different features having $\frac{1}{4}$, $\frac{1}{8}$, $\frac{1}{16}$, and $\frac{1}{32}$ of input image size. These multi-level extracted features are then passed into the MLP decoder to output segmentation of size $\frac{H}{4}\times \frac{W}{4}\times N_{Cats}$, where *N*_*Cats*_ denotes the number of categories requiring segmentation.

#### 2.1.1 Hierarchical transformer encoder

Mix transformer encoders (MiTs) have been used in hierarchical encoder. Multiple MiT encoders from MiT-B0, MiT-B2, MiT-B3, to MiT-B5, having the same architecture but different sizes, have been tried and evaluated. MiT-B0 is the lightest among all the least tuneable parameters and thus most efficient requiring lesser training and computational resources. MiT-B5, on the other hand, is the larger and more powerful model with greater number of tuneable parameters, as shown in Table 1, promising higher accuracy and performance at a higher computational cost.

**Table 1:** Default specifications of different MiT models.

**Model**	**Depth**	**Attention heads**	**Hidden layers**	**Parameters**
MiT-B0	[2,2,2,2]	[1,2,5,8]	[32,64,160,256]	3,319,292
MiT-B2	[2,2,2,2]	[1,2,5,8]	[32,64,160,256]	24,196,288
MiT-B3	[2,2,2,2]	[1,2,5,8]	[32,64,160,256]	44,072,128
MiT-B5	[2,2,2,2]	[1,2,5,8]	[32,64,160,256]	81,443,008

#### 2.1.2 Hierarchical feature representation

Multiple transformer blocks of hierarchical transformer encoder generate multi-level features from an input image. Each block takes a certain level of features as input and outputs a higher level of features. The model combines these features of different levels hierarchically and therefore can capture multi-scale context, enhancing its performance at semantic segmentation tasks. Patches generated from input image having size of *H*×*W*×3 are merged to generate hierarchical feature map *F*_*i*_ having resolution of $\frac{H}{2^{i+1}}\times \frac{W}{2^{i+1}}\times C_{i}$, where *i* ∈ {1, 2, 3, 4} and *C*_*i*+1_ are larger than *C*_*i*_.

#### 2.1.3 Overlapped patched merging

The process of merging patches for Segformer (36) is developed on the patch merging process of ViT (37) where a patch of *N*×*N*×3 is converted into a vector of 1 × 1 × *C*. The process can similarly be used to convert a feature map of 2×2×*C*_*i*_ into a 1×1×*C*_*i*+1_ vector to obtain a hierarchical feature map. The same method can be used to obtain feature map $F_{2}\left(\frac{H}{8}\times \frac{W}{8}\times C_{2}\right)$ from $F_{1}\left(\frac{H}{4}\times \frac{W}{4}\times C_{1}\right)$ and other features in the hierarchy. This is, however, important to highlight that the patch merging process for ViT (37) was non-overlapping which compromised local continuity around patches, whereas Segformer uses an overlapping patch merging process. This overlapping is achieved by introducing padding and a stride that is smaller than the size of the patch. For the sake of consistency with Segformer, we also use *K*, *S*, and *P* to denote patch size, stride between adjacent patches, and padding, respectively. We have used *K* = 7, *S* = 3, and *P* = 3 to generate overlapping patches to preserve continuity.

#### 2.1.4 Efficient self-attention

Transformer-based models require higher computational resources for the processing of high-resolution images because of the self-attention layer in encoders. As multi-head self-attention layer calculates self-attention as follows:


(1) $$\begin{array}{l} Attention\left(Q,K,V\right)=Softmax\left(\frac{QK^{T}}{\sqrt{d_{head}}}\right)V \end{array}$$

where *Q*, *K*, and *V* have the same dimension of *N*×*C*, where *N* = *H*×*W* is the length of sequence. The calculation of attention using Equation (1) results in computational complexity of *O*(*N*^2^) which restricts these models from being used for high-resolution images in given computational resources. To address the complexity issue and make the self-attention module efficient, the spatial reduction attention (SRA) layer is employed as proposed in the study mentioned in the reference (38). SRA efficiently reduces the sequence length of *K* and *V* before processing. The sequence reduction process for stage *i* is given as follows:


(2) $$\begin{array}{l} SRA\left(Q,K,V\right)=Concat\left(head_{0},...,head_{Ni}\right)W^{o} \end{array}$$

(3) $$\begin{array}{l} head_{j}=Attention\left(QW_{j}^{Q},SR\left(K\right)W_{j}^{K},SR\left(V\right)W_{j}^{V}\right) \end{array}$$

where *Concat*(·) is the concatenation. $W_{j}^{Q}\in {\mathbb{R}}^{c_{i}\times d_{head}}$, $W_{j}^{K}\in {\mathbb{R}}^{c_{i}\times d_{head}}$, $W_{j}^{V}\in {\mathbb{R}}^{c_{i}\times d_{head}}$, and $W^{o}\in {\mathbb{R}}^{c_{i}\times c_{i}}$ are linear projections. *N*_*i*_ denotes the number of heads for attention layer at *i*^*th*^ stage and thus dimension of each head, *d*_*head*_, is $\frac{C_{i}}{N_{i}}$. *SR*(·) is spatial reduction mechanism for input sequences *K* and *V* and is given as follows:


(4) $$\begin{array}{l} SR\left(\text{x}\right)=Norm\left(Reshape\left(\text{x},R_{i}\right)W^{S}\right) \end{array}$$

where $\text{x}\in {\mathbb{R}}^{H_{i}\times W_{i}\times C_{i}}$ is the input sequence, *R*_*i*_ is the reduction ratio at *i*^*th*^ stage, and *Reshape*(x, *R*_*i*_) reshapes input sequence to a size of $\frac{H_{i}\times W_{i}}{R_{i}^{2}}\times \left(R_{i}^{2}C_{i}\right)$. $W^{s}\in {\mathbb{R}}^{\left(R_{i}^{2}C_{i}\right)\times C_{i}}$ is the linear projection to reduce the size of the input sequence to *C*_*i*_, and *Norm*(·) denotes layer normalization.

The computational complexity, *O*(*N*^2^), of the self-attention mechanism is reduced to $O\left(\frac{N^{2}}{R_{i}^{2}}\right)$ as lengths of the sequences are now reduced using Equations (2)–(4). We have used *R* = [64, 16, 4, 1] in our experiment at each reduction stage.

#### 2.1.5 Mix-feed-forward network

Transformer-based models generally use positional encoding (PE) to capture spatial information regarding input features. The method causes deterioration in the performance of models for cases where the resolution of training images is different from test images. In such cases, PE, originally having a fixed resolution, is interpolated, resulting in reduced performance of the model. To address this issue, conditional positional encoding (CPE) (39) introduces a data-driven positional encoding generator (PEG) that can handle sequences that are longer than those used for the training of the model. PEG has been implemented by 2-D convolution with the kernel of size 3 × 3 in combination with PE. Based on the idea that CNN implicitly encodes positional information because of zero padding and border (40), the Segformer maintains that PE is not explicitly required for semantic segmentation tasks. Therefore, Segformer (36), capitalizing the idea of CNN capturing the positional information because of zero padding, employs Mix-FFN. We, therefore, use 3 × 3 convolution kernel for implementing Mix-FFN as follows:


(5) $$\begin{array}{l} X_{out}=MLP\left(GELU\left(Conv_{3\times 3}\left(MLP\left(X_{in}\right)\right)\right)\right)+X_{in} \end{array}$$

where *X*_*in*_ is the feature extracted by the efficient self-attention module. An MLP and 2-D convolution with a kernel of size 3 × 3 are combined in mix-FFN to generate output with positional information using Equation (5).

#### 2.1.6 Lightweight all MLP-decoder

The method employs a rather simpler and lighter decoder consisting of MLP layers. The decoder operates automatically without the need for hand-crafted features or manual tuning, and performs efficiently with fewer computational resources. The latent capability of the hierarchical transformer encoder to capture a larger effective receptive field (ERF) than CNN encoders complements the performance of the MLP decoder, enabling it to demonstrate higher performance.

The MLP decoder performs four distinct functions progressively as given by Equations (6)–(9). The first MLP layer takes multi-level features *F*_*i*_, extracted by the MiT encoder, as inputs and combines dimensions of their channels. Taking the output of the first layer, the second MLP layer up-samples features by $\frac{1}{4}$ and concatenates them together. The third layer fuses concatenated features as *F*, and the final layer generates a segmentation mask of size $\frac{H}{4}\times \frac{W}{4}\times N_{Cats}$, indicating *N*_*Cats*_ in segmented output. The decoder has been shown in Figure 1 and represented as follows:


(6) $$\begin{array}{l} \hat{F_{i}}=Linear\left(C_{i},C\right)\left(F_{i}\right),\forall_{i} \end{array}$$

(7) $$\begin{array}{l} \hat{F_{i}}=Upsample\left(\frac{W}{4}\times \frac{W}{4}\right)\left(\hat{F_{i}}\right),\forall_{i} \end{array}$$

(8) $$\begin{array}{l} F=Linear\left(4C,C\right)\left(Concat\left(\hat{F_{i}}\right)\right),\forall_{i} \end{array}$$

(9) $$\begin{array}{l} M=Linear\left(C,N_{Cats}\right)\left(F\right) \end{array}$$

- *M* in the equation represents the output segmentation mask,
- *Linear*(*C*_*i*_, *C*_*out*_)(·) refers linear layers with *C*_*in*_ and *C*_*out*_ as dimensions of input and output vectors, respectively.

## 3 Experiment/implementation

### 3.1 Data

The dataset provided by the study mentioned in the reference (41) containing 290 histopathology images and corresponding hand-annotated segmentation masks has been used. In total, 290 slides containing specific tissue sections which principally represented typical cases of non-melanoma skin cancer are part of the dataset. The dataset contains three types of non-melanoma skin cancer in varying proportions such that 140 slides of BCC, 60 slides of SCC, and 90 slides of IEC. Moreover, specimens have been extracted differently as it includes 100 specimens of shaved, 58 specimens of punched, and 132 specimens of excisional biopsies. The tissue sections which were most representative of cancer were indicated on each slide by pathologists. The data were collected over 4 months from late 2017 to early 2018. The ages of patients range from 34 to 96 years with a median of 70 years while the male to female proportion is 2:1. The images of biopsy samples were taken using high magnification and pre-processed so that each pixel of image saved as TIF represents 0.67 μm of tissue.

### 3.2 Segmentation and ground truth

**Figure 2:**
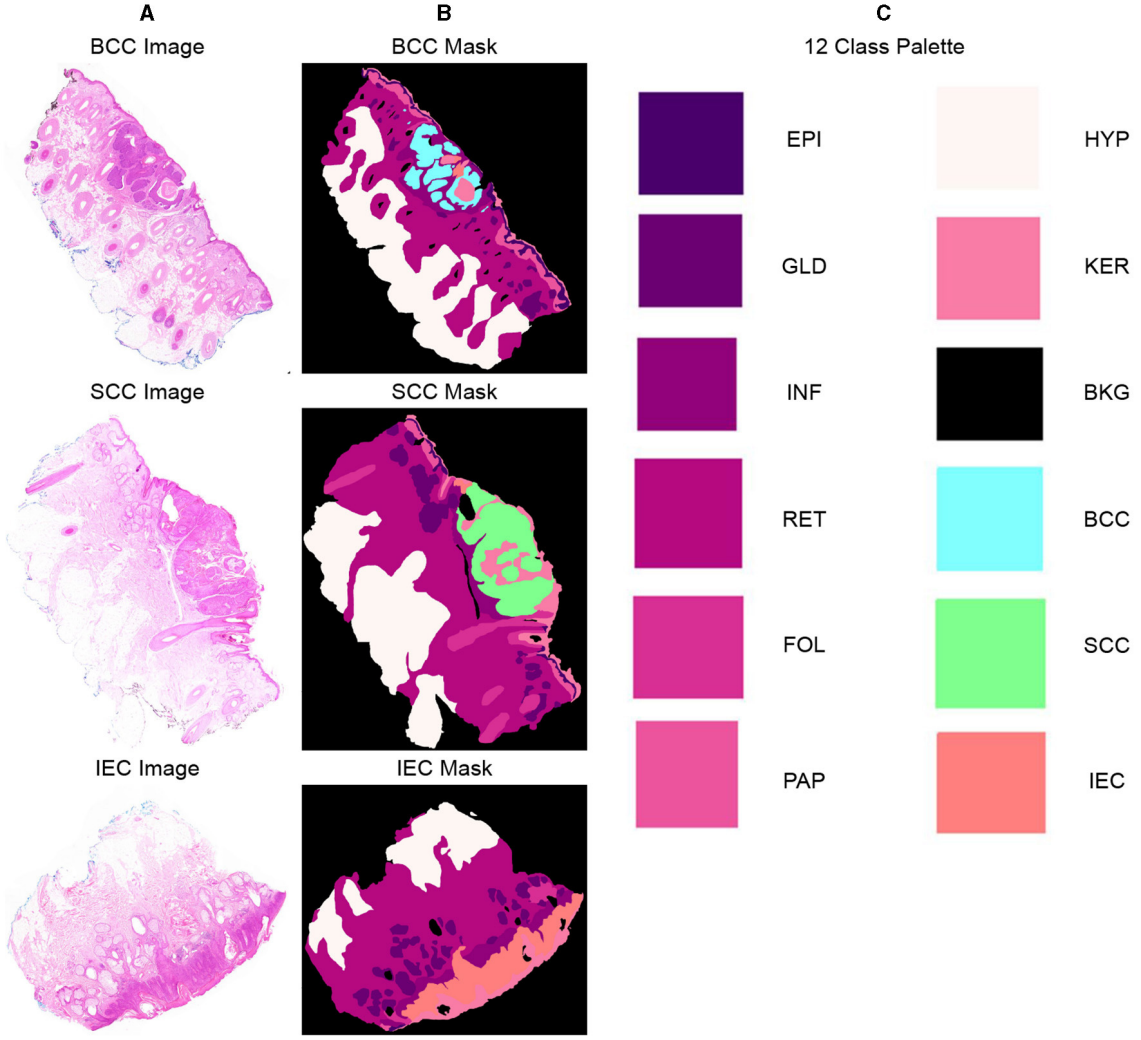
Representative samples and corresponding segmentation masks (ground truth) from the dataset, indicating 12 distinct classes and their corresponding color palette. **(A)** Sample histopathology images displaying instances of BCC, SCC, and IEC. **(B)** Corresponding segmentation masks indicating BCC, SCC, and IEC regions and other classes within the images. **(C)** List of all 12 classes, including BCC, SCC, and IEC, along with the corresponding color palette used for their visual representation.

Twelve classification categories, including carcinomas, were identified in specimen slides. These include BCC, SCC, IEC, Glands (GLD), Hair Follicles (FOL), Inflammation (INF), Reticular Dermis (RET), Hypodermis (HYP), Papillary Dermis (PAP), Epidermis (EPI), Keratin (KER), and Background (BKG). The pixel counts and percentage representation of these classes in the data are shown in Table 2. All these categories were painted with different colors over images using ImageJ for creating ground-segmentation truth. It is, however, highlighted that a perfectly healthy epidermis has been marked as EPI, and anomaly features such as dysplastic keratinocytes (solar keratosis) and carcinomas have been included in IEC. This ensures that variations in the epidermis that are non-cancerous but different from healthy epidermis are not wrongly classified. These segmentation masks have been created by trained professionals in consultation with pathologists in ~250 h and saved in PNG format, specifying each pixel as 1 of the 12 classes with a different color. These classes and color pallets are shown in Figure 2. The diligent work that put in to classify each pixel, on one hand, ensures the accuracy of masks while, on the other hand, it brings conceptual and implementation challenges. While evaluating slides for distinguishing class boundaries, pathologists work on slides at different magnification levels. In addition, they work on different conceptual levels, depending on competence and previous exposure based on experience. This is necessitated as boundaries between certain tissues such as the basement membrane between epidermis and papillary dermis layers are only distinguishable at high magnifications, whereas boundaries between some classes such as papillary and reticular dermis are even more diffused and indistinguishable at lower resolutions (32).

**Table 2:** Distribution of pixels and percentage representation of classes in the dataset.

**Classes**	**Pixels**	**Representation**
FOL	675,714	0.20%
GLD	1,379,800	0.40%
EPI	2,276,658	0.60%
IEC	3,527,541	1.00%
BCC	3,984,910	1.10%
PAP	5,965,621	1.70%
KER	7,438,263	2.10%
SCC	8,778,154	2.40%
INF	10,120,508	2.80%
HYP	33,716,745	9.30%
RET	71,645,463	19.80%
BKG	211,444,704	58.60%

**Figure 3:**
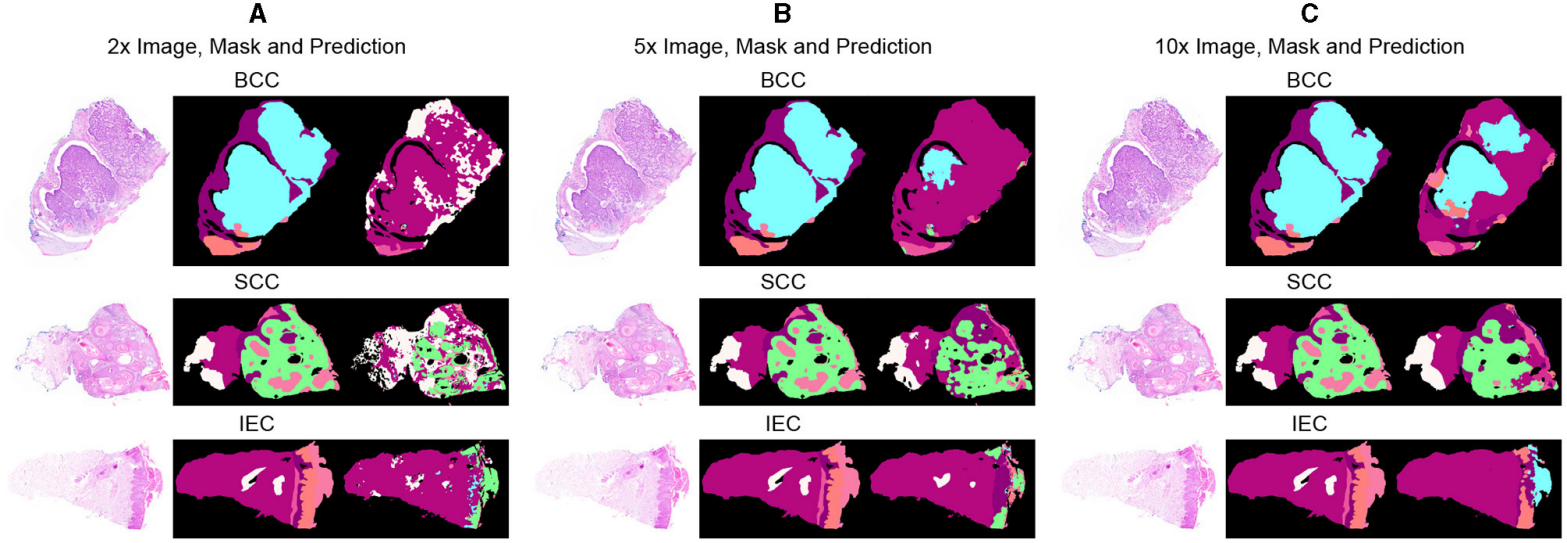
Comparative analysis of specimen images, segmentation masks, and corresponding predictions at three different resolutions. **(A)** Sample images of BCC, SCC, and IEC at the resolution of 2x, alongside their corresponding segmentation masks and the predictions generated by the segmentation model. **(B)** Sample images of BCC, SCC, and IEC at the resolution of 5x, alongside their corresponding segmentation masks and the predictions. **(C)** Sample images of BCC, SCC, and IEC at the resolution of 10x, alongside their corresponding segmentation masks and the predictions.

Segmentation accuracy is commonly measured on a pixel basis and is also contingent upon the resolution of images. As magnification can influence the accuracy of a pathologist, similar resolution of images in the dataset can also influence the performance of an algorithm. To validate this point, all images are down-sampled by a factor of 2, 5, and 10 and saved as 2x, 5x, and 10x, respectively. As shown in Figure 3, a critical visual study of multiple images and masks reflects that no discernible difference was observed in original resolution and at 2x as logically understandable, and certain features and their boundaries became indistinguishable at 10x.

### 3.3 Experimental setting and training

We implemented Segformer with Python using the platform of hugging face. The model was trained using Dual NVIDIA GeForce RTX 2070 of 8 GB. We arranged training in batches of size 8 with 50 epochs using Adam optimizer and a learning rate of 0.0006. We used layer normalization with 1 × 10^−6^ to achieve better generalization considering our smaller batch size. We used the Gaussian error linear unit (GELU) activation function for hidden layers of the decoder and used a dropout rate of 0.1 to keep the model from over-fitting. The data were randomly split into training, validation, and test sets with a proportion of 80, 10, and 10%, respectively. A deliberate effort was made to distribute all types of cancers and biopsies evenly in each split. The overlapping patches were created using *K* = 7, *S* = 3, and *P* = 3. The original patch size of 256 × 256 was re-scaled later to 512 × 512. Moreover, to overcome the scarcity of our data, we incorporated data augmentation and assess the sensitivity of our model toward the resolution of data, and we separately used three different resolutions 2x, 5x, and 10x for validation and testing after training on 2x.

## 4 Results and discussions

### 4.1 Initial results

We evaluated the performances of the segmentation models through three different commonly used performance measures: F1 score, mean intersection over union (mIoU), and average accuracy. Initially, we performed experiments with four different segmentation models, namely, MiT-B0, MiT-B2, MiT-B3, and MiT-B5. All these fine-tuned models were then trained on a 10x dataset and later validated and tested on validation sets and test sets of not only corresponding 10x but on 2x and 5x resolutions also. The segmentation accuracy achieved by both untrained and pre-trained models is shown in Table 3 for all resolutions.

**Table 3:** Performance measures of MiT models with and without pre-training for data resolutions of 2x, 5x, and 10x.

**Data resolution**	**Model**	**Pre-trained**	**F1 score**	**mIoU**	**Average accuracy**
2x	MiT-B0	False	0.844	0.402	0.518
	MiT-B0	True	0.867	0.609	0.712
	MiT-B2	False	0.782	0.381	0.487
	MiT-B2	True	0.868	0.587	0.746
	MiT-B3	False	0.827	0.371	0.472
	MiT-B3	True	0.869	0.624	0.761
	MiT-B5	False	0.779	0.305	0.409
	MiT-B5	True	0.881	0.639	0.731
5x	MiT-B0	False	0.868	0.428	0.536
	MiT-B0	True	0.899	0.627	0.745
	MiT-B2	False	0.808	0.399	0.500
	MiT-B2	True	0.897	0.604	0.762
	MiT-B3	False	0.840	0.392	0.491
	MiT-B3	True	0.893	0.640	0.772
	MiT-B5	False	0.803	0.327	0.428
	MiT-B5	True	0.907	0.651	0.762
10x	MiT-B0	False	0.873	0.435	0.546
	MiT-B0	True	0.907	0.631	0.754
	MiT-B2	False	0.852	0.404	0.507
	MiT-B2	True	0.909	0.630	0.770
	MiT-B3	False	0.846	0.400	0.498
	MiT-B3	True	0.905	0.642	0.780
	MiT-B5	False	0.812	0.330	0.441
	MiT-B5	True	0.911	0.654	0.774

Among datasets of different resolutions, the highest accuracy has been achieved by the dataset of 10x resolution with F1 score, mIoU, and average accuracy of 0.911, 0.654, and 0.780, respectively. Moreover, the highest performance scores among all models have been achieved by the pre-trained MiT-B5 with 10x dataset. It achieved an F1 score of 0.911 and mIoU of 0.654, whereas the highest average accuracy of 0.780 was achieved by pre-trained MiT-B3 on the same dataset. The variation can be attributed to the ways, and these performance measures are calculated and affected by the distribution and proportion of classes and background pixels in test samples.

Table 3 further reveals correlations between data resolution, pre-training, model size, and performance measures. It can be observed that an increase in data resolution results in decreased performance for all models. An explanation for this can be sought by keenly observing specimens of various resolutions and predictions, as shown in Figure 3. It is obvious to notice that while moving from Figures 3A–C, predictions are getting better and more similar to ground truths. Seeing predictions from high resolution samples, one can suggest that additional detail in high resolution samples acts as noise, thus confusing the model and resulting in noisy predictions and deteriorated performance. The trend also implies that there is sufficient information in 10x images to enable the model to predict accurately. In addition, all models may be performing better with 10x data than others because they are trained on 10x, whereas they are being tested on 2x and 5x. Moreover, considering the computational resources required for training and processing high resolution images and desired results, it can be assumed that image resolution equivalent to 10x data may deliver excellent results for most applications, and therefore, our further study is based on data of 10x resolution.

It is also noticeable that the introduction of pre-training in MiT-B0 results in an increase of more than 38% in average accuracy. Moreover, the trend is similar to F1 score and mIoU across all models. It is also noticed that performance measures consistently improve from model MiT-B0, MiT-B2, MiT-B3 to MiT-B5. We notice that the accuracy for 10x data increases from 75.4% for pre-trained MiT-B0 to 77.4% for pre-trained MiT-B5. It is, however, highlighted that the gain in accuracy from MiT-B0 to MiT-B5 is 2%, whereas the number of tuneable parameters increased from 3,319,292 to 81,443,008; a tremendous increase would require enormous computational and training resources. This leaves us with a compromise between real-time fast processing or high performance—we may settle for MiT-B0 while needing fast processing compromising sightly on performance or use MiT-B2 or MiT-B5 for best performance. We further highlight that MiT-B0 has been used for detailed experiments in our study.

### 4.2 Ablation studies

#### 4.2.1 Effect of change in model depth

As already highlighted in Table 1, all the MiT models under discussion have a default depth of [2,2,2,2]. We experimented with MiT-B0 by changing the default depth to [1,1,1,1] and [4,4,4,4]. We observe that a change in depth from default to [1,1,1,1] and [4,4,4,4] in a pre-trained model does not have any positive impact. The average accuracy decreases from 75.42%, in the case of the default model, to 72.12 and 73.10% for models with depths of [1,1,1,1] and [4,4,4,4], respectively. The trend is similar for the F1 score and mIoU.

#### 4.2.2 Effect of change in the number of attention heads

The accuracy of MiT-B0 declines negligibly to 74.94% when attention heads are changed to [1,1,2,4] from the default of [1,2,5,8]. The same number, however, increases to 77.1% when attention heads are set to [2,4,10,16], yielding an increase of 2% from default. Similarly, the F1 score and mIoU also decrease in the former case and increase when attention heads are set to [2,4,10,16]. It is also important to note that this combination results in the best accuracy, as shown in Table 4. This model, therefore, has been further studied and evaluated in succeeding paras and finally proposed for multi-class segmentation.

**Table 4:** Ablation studies with modifications in pre-trained MiT-B0.

**Model**	**Modification**	**F1 score**	**mIoU**	**Average accuracy**
**Modification in model depth**				
MiT-B0	Depth [2,2,2,2]^*^	0.907	0.631	0.754
	Depth [1,1,1,1]	0.900	0.589	0.721
	Depth [4,4,4,4]	0.905	0.595	0.731
**Modification in attention heads**				
MiT-B0	Attention Heads [1,2,5,8]^*^	0.907	0.631	0.754
	Attention heads [1,1,2,4]	0.906	0.618	0.749
	Attention heads [2,4,10,16]	0.909	0.643	0.771
**Modification in hidden layers**				
MiT-B0	Hidden layers [32,64,160,256]^*^	0.907	0.631	0.754
	Hidden layers [16,32,80,128]	0.889	0.505	0.623
	Hidden layers [64,128,360,512]	0.895	0.536	0.655

Default parameters are indicated with the symbol of *.

#### 4.2.3 Effect of change in hidden layers

We experimented with hidden layers of the model both by decreasing them to [16,32,80,128] and also by increasing them to [64,128,360,512] from the default layers of [32,64,160, 256]. As shown in Table 4, the change in hidden layers from default does not improve accuracy, F1 score, or mIoU. It is, however, noticeable that the model with hidden layers [16,32,80,128] shows a massive decrease of 17% in accuracy dropping it to 62.3%.

#### 4.2.4 Introduction of data augmentation

We have already ascertained that pre-trained MiT-B0 performs better than one that is not pre-trained. We have also tried different modifications in MiT-B0 and listed corresponding performance measures, as shown in Table 4. The results show that pre-trained MiT-B0 performs the best when attention heads are [2,4,10,16]. We, now, introduce data augmentation to our selected models to evaluate its impact on segmentation performance measures. We augmented data by flipping original images and later rotating each of the original and flipped images by 90,180, and 270 degrees, replacing every original image with eight images in the augmented dataset. We assume that data augmentation using this technique would also make the model rotation invariant. The segmentation performance measures of different pre-trained models after data augmentation are shown in Table 5. It can be observed that the average accuracy for pre-trained default MiT-B0 increases by ~4%, reaching 78.5%. Similarly, ~2% increase is observed in mIoU, which improves from 0.631 to 0.644, whereas no change is observed in the F1 score. The best performing proposed MiT-B0, as evaluated in Table 4, also performed better with data augmentation as its average accuracy peaks to 83.1% from 77.1%; the mIoU also improves to 0.653 from 0.643, whereas F1 score negligibly declines. This experiment promises better and improved performance of the model as more data are accumulated and used for training.

**Table 5:** Performance measures of pre-trained MiT-B0 models with data augmentation.

**Model**	**Data augmentation**	**Modification**	**F1 score**	**mIoU**	**Average accuracy**
MiT-B0	False	None	0.907	0.631	0.754
	True	None	0.907	0.644	0.785
	True	Attention head [2,4,10,16]	0.908	0.653	0.831

### 4.3 Quantitative results

We present performance measures for pre-trained default MiT-B0 and our proposed modified MiT-B0 with data augmentation, as shown in Table 6. We also tabulate the results of the study mentioned in the reference (32) in the same table for comparative analysis. The table shows that the proposed model achieved an average accuracy of 83.1%, which is significantly 4% higher than the study mentioned in the reference (32). The proposed model achieved an F1 score of 0.908 and mIoU of 0.653.

**Table 6:** Performance measures of models in comparison; all models are pre-trained and data augmented.

**Model (Pre-trained and data augmented)**	**F1 score**	**mIoU**	**Average accuracy**
Thomas et al. (32)	–	–	0.799
MiT-B0 (default)	0.907	0.644	0.785
MiT-B0 (proposed)	**0.908**	**0.653**	**0.831**

The highest performance measures are highlighted as bold.

Moreover, Table 7 presents the classwise accuracy of models in comparison. It is noticeable from the table that no single model can be declared a categorical winner in classifying all classes more accurately than others. The proposed MiT-B0 classified seven classes more accurately than the study mentioned in the reference (32), whereas the later one was more accurate in predicting five classes. Moreover, the performance of default MiT-B0 and the study mentioned in the reference (32) was equal for the class GLD with an accuracy of 87.3%. The proposed model demonstrated a significant increase in performance that ranges from a minimum of 3% to a maximum of 30%. The highest increase in performance is 30% which is observed in the case of RET, where the proposed model achieved an accuracy of 91.2%. The minimum increase of 3% is observed in recognizing GLD, where the proposed model achieved an accuracy of 89.7%. The leading accuracy was achieved by the proposed model in recognizing BKG, HYP, and BCC with a score of 98.7, 93.5, and 91.5%, respectively. The lowest accuracy of 65.8% is observed in identifying FOL, which is still ~7% higher than the study mentioned in the reference (32). The other two categories with lower accuracy of 71.5 and 74.8% are INF and PAP, respectively. It is important to highlight that performance of the proposed model is 25% higher than the study mentioned in the reference (32) in the case of INF, whereas it is 7% lower in the case of PAP. It is noticeable that lower results of FOL, INF, and PAP are degrading the overall performance and causing lower accuracy. The lower performance for FOL, INF, and PAP may be attributed to their lower representation in the dataset as the proportion of their pixels is 0.2, 2.8, and 1.7%, respectively. The cause of lower results in the case of FOL can also be traced by exploring the way pathologists identify FOL by evaluating tissues at higher levels rather than studying their high-resolution microscopic structures (32).

**Table 7:** Classwise segmentation accuracy of models in comparison.

**Model**
**(Pre-trained and data augmented)**	**BKG**	**BCC**	**SCC**	**IEC**	**EPI**	**GLD**	**INF**	**RET**	**FOL**	**PAP**	**HYP**	**KER**
Thomas et al. (32)	0.95	0.865	**0.857**	0.707	**0.831**	0.873	0.574	0.702	0.615	**0.808**	**0.962**	**0.846**
MiT-B0 (default)	0.983	0.905	0.707	0.787	0.734	0.873	0.692	0.909	0.558	0.646	0.869	0.757
MiT-B0 (proposed)	**0.987**	**0.915**	0.786	**0.814**	0.791	**0.897**	**0.715**	**0.912**	**0.658**	0.748	0.935	0.813
Percentage difference	**4%**	**6%**	-8%	**15%**	-5%	**3%**	**25%**	**30%**	**7%**	-7%	-3%	-4%

The highest accuracy for each class is highlighted as bold. The percentage difference in fourth row has been calculated considering accuracy achieved by Thomas et al. (32) as original and by proposed model as the new one therefore, all the positive values which are highlighted as bold as well, indicating classes for which proposed model has performed better than others.

**Figure 4:**
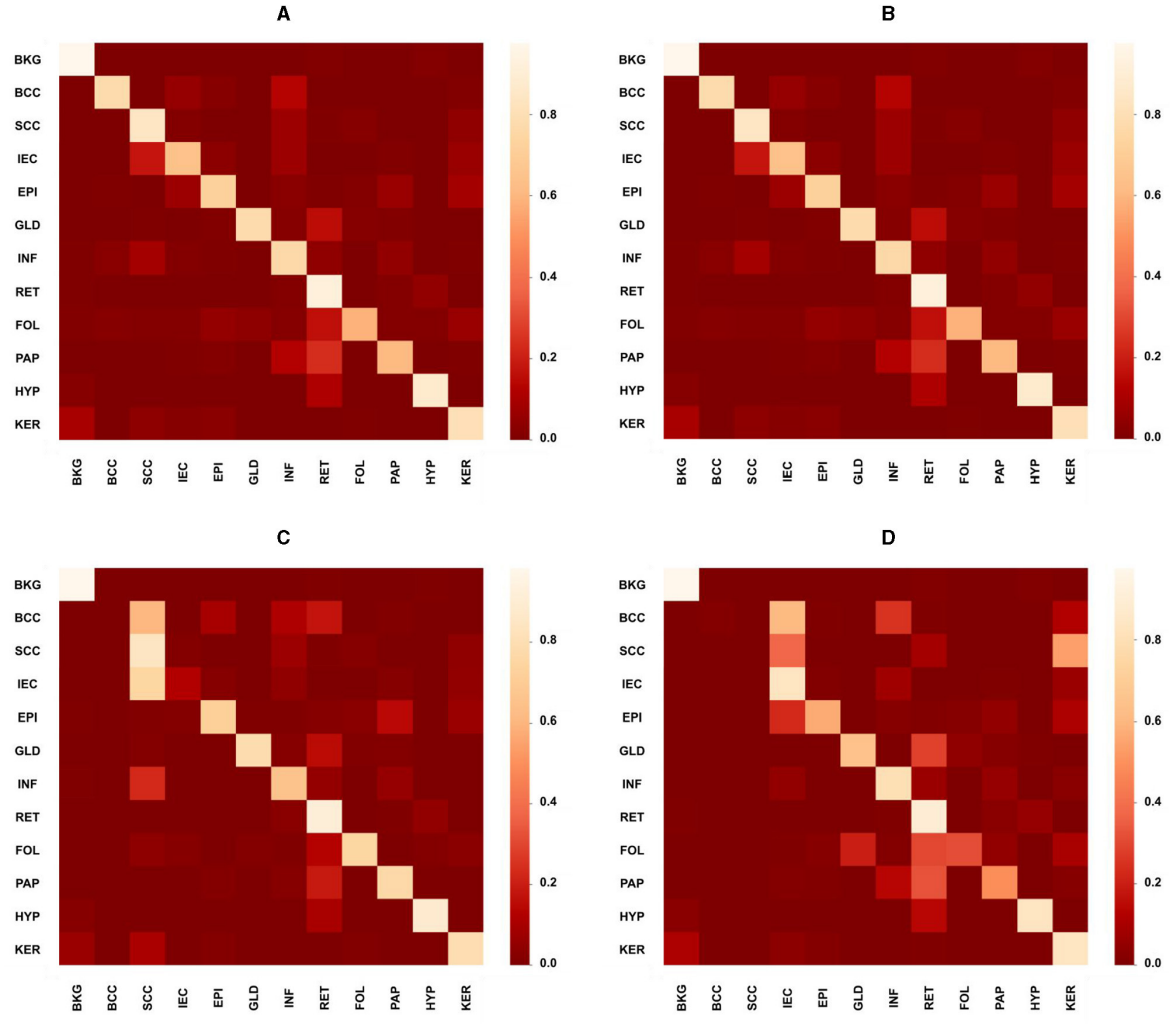
Confusion matrices presenting the performance of the proposed model across different test cases. **(A)** Overall confusion matrix presenting the performance of the proposed model for test cases of all carcinomas, i.e., BCC, SCC, and IEC. **(B)** Confusion matrix presenting the performance of the proposed model for BCC test cases only. **(C)** Confusion matrix presenting the performance of the proposed model specifically for the SCC test case. **(D)** Confusion matrix presenting the performance of the proposed model specifically for the IEC test case.

While the proposed model performed considerably better than the study mentioned in the reference (32) in most cases with an average increase in accuracy nearing 13%, however, in the case of SCC, EPI, PAP, HYP, and KER, it did not perform well where the average decrease is 5%. The proposed MiT-B0 achieved an accuracy of 78.6% in comparison to 85.7% in the case of SCC. Similarly, its score was 79.1, 74.8, 93.5 and 81.3% for EPI, PAP, HYP, and KER, respectively. The leading difference of 8, 7, and 5% is observed in SCC, PAP, and EPI, respectively, in comparison to the study mentioned in the reference (32). The lower performance in the case of SCC may be a result of under representation as there are only 60 images of SCC with a pixel proportion of 2.4%. Figures 4A–D presents the confusion matrices of the proposed model across different test cases and a critical analysis of Figures 4C, D indicates that there is a higher degree of confusion between these two cases, and therefore, these are classified relatively less accurately. The model, however, predicted IEC cases better because of their higher representation.

### 4.4 Qualitative results

**Figure 5:**
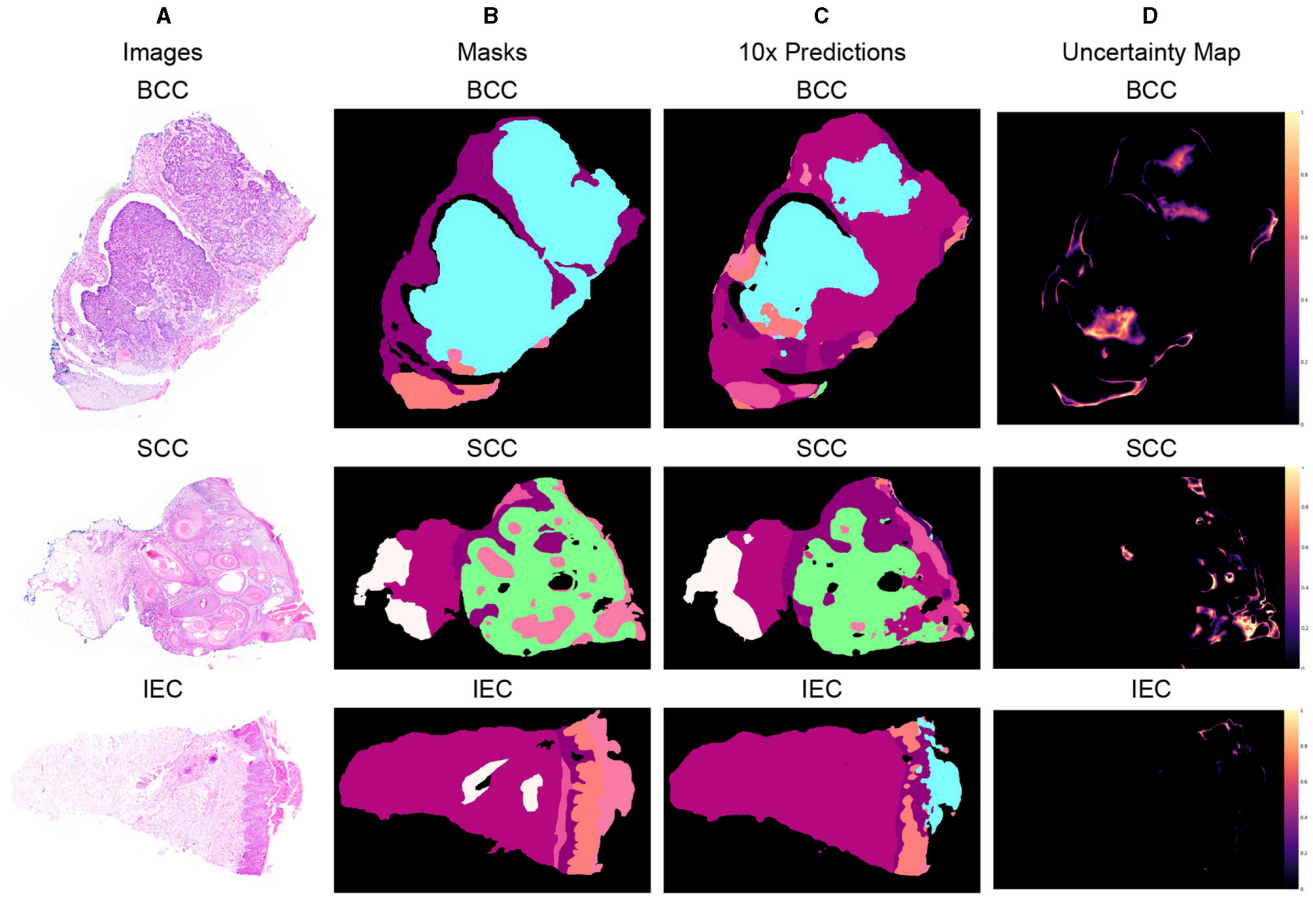
Segmented predictions by the model for test cases of BCC, SCC, and IEC along with visualization of the model's confidence in its predictions through uncertainty maps. **(A)** Sample images of BCC, SCC, and IEC. **(B)** Ground truth segmentation masks corresponding to the sample images. **(C)** Segmented outputs generated by the network for the test cases of BCC, SCC, and IEC. **(D)** Uncertainty maps indicating the model's confidence in its predictions.

Three different data samples of 10x resolution, corresponding segmentation masks, predictions, and uncertainty maps are shown in Figure 5. The critical visual analysis of segmentation masks and predictions reveals that both are greatly similar and thus confirm the results, as presented in Tables 6, 7. The classes such as BCC and IEC, which seem to be correctly classified in visual inspection, have higher accuracy, whereas classes such as FOL and INF seem to be classified less accurately.

The uncertainty maps have also been generated to visually present the confidence of the proposed model in its prediction. The concordance between expected accuracy, that is confidence of the model in prediction, and accuracy is highly desirable. Therefore, temperature-scaling of output was carried out for calibration (42). The uncertainty maps have been generated after achieving agreement in confidence and accuracy by subtracting the maximum class probability of each class from unity. It is important to highlight that the uncertainty, being referred to the current scenario, is not the degree of variance. These uncertainty maps point toward the areas where the model is not very confident in its prediction and visual analysis indicates that boundaries between classes and tissues such as FOL, INF, and PAP are the main areas of uncertainty. Furthermore, the areas of higher uncertainty highlight areas of higher complexity involving multiple tissue classes and boundaries. It can be assumed that these complex areas could be handled better provided images of higher resolutions such as 2x or 5x and are used for training and predictions but definitely at a much higher cost. It is also worth mentioning that the visualization of uncertainty maps not only qualitatively confirms our analytical results but it is also an interpretable way of demonstrating performance, which could enhance the trust of pathologists and clinicians in the segmentation model.

Furthermore, after critical evaluation of Figure 3, it can be safely inferred that no discernable difference can be observed with the naked eye in data samples of higher resolution when compared with lower ones. Analysis of segmentation predictions generated by different resolutions however reveals that they are different from each other and some from ground truth as well. Predictions from high resolution samples are not as close to segmentation masks as are low resolution predictions. This is also shown in Table 3. Moreover, high resolution predictions are more noisy than lower ones which can be attributed to the ability of the model to capture fine details and present them in predictions. Predictions also indicate the ability of the model to distinctly identify the boundaries between tissue types.

## 5 Conclusion

Our study manifested that non-melanoma skin cancer comprising BCC, SCC, and IEC, which is more than 98% of all skin cancers, can be segmented into tissues and carcinomas by a fully automated transformer-based semantic segmentation framework. Since the segmentation model was trained using the lowest resolution 10x dataset, it is evident that promising segmentation results can be achieved with low resolution histopathology images. It has also been demonstrated that our framework performed fairly well when tested with samples of higher resolutions of 2x and 5x, despite being trained on a lower resolution dataset proving its robustness. Moreover, it is also important to highlight that these results have been achieved for the segmentation of 12 different classes with a very limited number of samples. Therefore, the excellent performance of the proposed segmentation model with a fair degree of certainty implies a great potential which can be capitalized for the automatic detection and classification of skin cancer cases through transformer-based AI frameworks.

While our study primarily focuses on achieving high segmentation accuracy, we recognize the importance of computational efficiency in practical settings. Our findings emphasize the need to strike a balance between accuracy and computational resources. Through the modified variant of Segformer, we demonstrate the model's ability to effectively handle the complexities of histopathology image analysis while maintaining computational efficiency. Moreover, it is also essential to highlight the limitations of our study. First, our comparison with interpretable models is limited to only one benchmark, as there is a lack of published results from studies employing interpretable methods on the same dataset. This constraint highlights the need for future research to explore additional interpretable models and conduct comprehensive comparisons. Second, our model's performance was suboptimal for certain classes, such as PAP, HYP, and KER, which have lower representation in the dataset. This limitation underscores the importance of addressing class imbalance issues in future studies, such as through data augmentation techniques or specialized training strategies.

In light of the above-mentioned limitations, future research recommendations include further investigation of interpretable models for skin cancer segmentation, exploring techniques to mitigate class imbalance effects and enhancing the computational efficiency of segmentation frameworks. By addressing these challenges, we aim to advance the field of automated skin cancer diagnosis and contribute to improved patient care and outcomes.

## Data availability statement

The original contributions presented in the study are included in the article/supplementary material, further inquiries can be directed to the corresponding author.

## Author contributions

MI: Data curation, Formal analysis, Methodology, Resources, Visualization, Writing – original draft. MIT: Supervision, Validation, Writing – review & editing. MM: Data curation, Methodology, Software, Visualization, Writing – original draft. NA: Funding acquisition, Methodology, Visualization, Writing – review & editing. MA: Formal analysis, Methodology, Project administration, Supervision, Writing – review & editing.
